# Growth retardation among children in southern Iran: a 7-year population based cohort study

**DOI:** 10.1186/s12889-020-09511-w

**Published:** 2020-09-11

**Authors:** Mohammad Javad Fatemi, Mostafa Dianatinasab, Golnaz Sharifnia, Hossein Moravej, Mohammad Fararouei

**Affiliations:** 1 Student research center for health sciences, department of epidemiology, school of health, Shiraz University of Medical Sciences, Shiraz, Iran; 2 Center for Health Related Social and Behavioral Sciences Research, Shahroud University of Medical Sciences, Shahroud, Iran; 3 Department of Epidemiology, School of Public Health, Shahid Sadoughi University of Medical Sciences, Yazd, Iran; 4 Research center for health sciences, department of pediatrics, School of Medicine, Shiraz University of Medical Sciences, Shiraz, Iran; 5 Shiraz HIV/AIDS Research Center, Institute of Health, Shiraz University of Medical Sciences, Shiraz, Iran

**Keywords:** Growth retardation, Children, Height, Breastfeeding

## Abstract

**Background:**

Growth retardation is a common health problem, which requires early prevention and detection. This study was conducted to define the approximate age at which stunting starts among the Iranian boys and girls.

**Method:**

The second phase of a population-based retrospective cohort nested case-control study on 400 children who were followed from birth to 7 years of age. This study was performed to define the pattern of growth among stunted and normal children and to reveal the age at which stunting starts in each gender.

**Results:**

Of the selected participants, 53% were girls. Also, about 18% of the children registered by the selected health centers were defined as stunted (under the 3rd percentile of the corresponding sex-age NCHS/WHO growth reference). For boys, the height was relatively similar between the two groups until the age of 6 months at which the difference in height between normal and stunted children starts to become significantly large (difference = 0.70 cm, *P* = 0.04). For girls, height in the two groups is relatively similar until the age of 9 months at which the difference starts to become significantly large (difference = 0.97 cm, *P* = 0.01). No significant difference in the weight of the girls was observed between the normal and stunted groups during the study period (difference = 283.21 g, *P* > 0.05). However, boys from the stunted group were lighter since almost the same time that they started to become significantly shorter (difference = 1265.19 g, *P* = 0.001).

**Conclusions:**

Soon after birth (at about the 6 months of age), the growth pattern of some (stunted) children starts to stumble and divert from normal. The sixth month of age is the age at which mothers start weaning with withdrawing breast milk and start supplementary foods and adult diet. A specially designed study is needed to understand the actual reason for observing such a phenomenon among Iranian children.

## Background

Anthropometric indexes in early childhood are considered important measures when assessing children’s health [1]. For example, growth indices (in particular weight and height), that are widely used to assess children’s nutritional status [2], are associated with the risk of morbidity and mortality and future cognitive status of a person [3–7]. Growth charts are adequately precise, and yet easily applied, tools in evaluating the growth status of an individual [8]. Hight is considered as a good indicator of health in general when compared to weight. This is because height is a good proxy retrospective indicator of a child’s health status, including current or past conditions. Although there many definitions for suboptimal growth in children, height shorter than 5^th^ percentile or two standard deviations below the mean value of a population’s height is considered as growth failure [1]. suboptimal growth in children is common in the world. However, reports suggest that the condition is far more prevalent in low-income countries. In fact, it has been estimated that less than 50% of children under the age of 5 can catch-up to normal height in several low-income countries [2]. Short stature, without apparent pathologic condition, is considered as idiopathic short stature (ISS) [6]. Growth retardation often occurs so gradually that it is noticed by parents or health providers with a long delay. The delay is commonly so long that the condition cannot be properly and timely managed. In addition to weight and height, other indexes are also used for different proposes. For example, head circumference is an important index that may reflect an individual’s cognitive development [9, 10].

Every person doesn’t follow the same growth path as he/she has different growth potentials driven by genetic and environmental (e.g. health, socio-economic, and nutrition conditions) factors [6]. However, rather than genetic diversity, the observed differences in children’s stature between developed and developing countries are mainly associated with socio-economics and nutritional factors [11]. As a result, the justification of observed differences in children’s growth based on genetic diversity is not supported by the world health organization as it condemns the use of national growth standards in defining the growth status of a nation. According to WHO, young children with appropriate living conditions, achieve their optimum growth potentials irrespective of their genetic backgrounds [8]. A large number of studies reported several non-genetic determinants of stature in different countries [1, 6, 11, 12]. It seems that the determinants of growth failure (including ISS) are undefined for a significant proportion of cases. However, dietary factors are suggested to act as important contributors in developing countries [13, 14]. The first phase of this study introduced several contributing factors associated with stunting [14]. However, neither our previous report nor the existing literature, clearly stated the timing of the effect of the contributing factors (e.g. nutrition) on linear growth (i.e. at what age after birth the growth retardation starts) [15, 16]. By collecting extra information on anthropometric measures at different ages of the participants, we studied the timing of the start of stunting among boys and girls to see if there is any critical point for detection and prevention of growth retardation.

## Methods

### Settings

The present study was reported based the Strengthening the Reporting of Observational Studies in Epidemiology (STROBE) statement [17]. This is the second phase of a retrospective cohort nested case-control study on stunting and its related factors. The methods of sampling and data collection are previously discussed in detail [14]. Briefly, the study was conducted on 400 children who were registered with Shiraz healthcare centers and were followed from birth to seven years of age. Health centers in Iran provide primary health care including maternity and pregnancy care, vaccination, and monitoring children’s growth and development to the residences who are living in their defined geographical areas. The routine services for under seven years old children are mandatory and precisely scheduled.

### Sampling

More details on the methods of sampling is available elsewhere [14]. In brief, a total of 27000 children were registered with Shiraz health centers from 2009 to 2010. A list of all health centers was obtained from Shiraz University of Medical Sciences (the deputy of health), and 24 health centers were selected randomly. A research team member visited the selected health centers and obtained a list of registered children with 6-7 years of age (*n*=1570). The selected age range was defined to exclude puberty growth (puberty may occur before the age of nine in some girls). Also, anthropometric measures are not available for older children as they no longer have to visit health centers. Of the registered children, those with the interested age with normal weight and height at birth and completed their routine visits to the health centers were selected for the study, and height at the age of 7 was used to define growth retardation (ISS) among selected children.

### Data collection

A data collection form was used to extract data from the family’s health files. The extracted information included child’s gender and date of birth, measures of height (cm), weight (gr) and head circumference (cm) from birth to 7 years of age (i.e. at birth and months 2, 4, 6, 9, 12, 24, 36, 48, 60, 72 and 84), key nutritional information (i.e. starting and ending dates of breastfeeding, starting date and types of complementary foods), parent’s education and occupation, number of siblings, the birth interval of the child, type of delivery and presence of any health problem. Mothers of selected children were later contacted by the female health nurse and via a phone interview, the rest of the required information was obtained. During the phone interview, the mothers first received a brief explanation about the research as a study on growth determinants and a verbal informed consent was obtained from them. To control recall and reporting biases, mothers were not told why they were selected (their child is a case or control). The information collected from the phone interview included, any medical condition the child has or she/he had suffered in the past. The mothers also asked to answer a few key questions with regard to the child’s nutrition. The latest job and educational statuses of the parents were also reported by the mothers during the interview.

More details on the inclusion and exclusion criteria of this study have been described in our previous paper [14]. Briefly, all children who were between 6 and 7 years of age, were all born with a height upper than the 20^th^ percentile of the NCHS growth chart, were living with their both parents (based on mother’s report), were eligible to be included in the study. Children were also excluded if their mothers reported having changed their address as it could have caused a significant alteration in the child’s living and health condition (e.g. moving from rural areas to the city during the participant’s life).

### Selection of cases and controls

While extracting data from the selected families’ health files, the WHO’s definition for stunting was used to define stunting among the eligible children. Accordingly, among the 1570 children registered with the health centers 282 children (prevalence=17.96%) met the defined criteria of short for age (i.e. a height shorter than the 3^rd^ percentile of NCHS/WHO growth reference of children with the same sex at age 7) among which 200 children were selected as cases. Controls were selected randomly among children with a height taller than 20^th^ percentile of the same sex and age (7 years old) with completed routine visits to the health centers [14]. Both case and control participants were selected if a successful phone interview was made to mothers to acquire required information)

### Statistical methods and sample size

Post-hoc power analysis suggested that the sample size of the first phase of this study (200 stunted participants as cases and 200 controls with normal height at age 7) was adequate to obtain a significant difference in height between stunted and normal groups at different ages as small as 1 cm. In that regard, type one error and the power of the test were set to %5 and 80% respectively. The data was analyzed using SPSS statistical software, version 19. Independent sample t-test was used to define any significant difference in weight, height and head circumference between stunted and normal groups at different ages.

## Results

The demographic and anthropometric status of the stunted and normal groups are reported elsewhere [14] and not presented here to save the limited space. Briefly, of the children registered to the selected health centers, about 18% were defined as stunted (under 3^rd^ percentile of the corresponding sex-age NCHS/WHO height growth reference). Of the total sample, 53.0% were females (53.5% and 52.5% of the case and control groups were females respectively, *p*=0.85). No significant difference observed when the mean duration of breastfeeding was compared between females and males in the case (21.73 and 21.86 months respectively, *p*=.43) or control groups (21.94 and 22.36 months respectively, *p*=.59) using Mann-Whitney U test. In addition, of the case and control groups, 59% and 57% of fathers were unemployed respectively (*P*=0.003). Whereas, 83.5% and 88.5% of the mothers from case and control groups were homemakers respectively (*P*=0.15). Concerning income, 17.0% and 36.5% of the participants’ families of case and control groups had an income of more than 30 million Rials respectively (*P*<0.001). With regard to the parent’s education, both fathers and mothers of the participants in the control group were more educated (*P*<0.001).

As presented in Table 1, the comparison of height, weight and head circumference at birth among boys shows no significant difference between the stunted and normal groups. The observed similarity in height between the stunted and normal groups remains relatively unchanged until 6 months of age (difference=0.70 cm, CI95%= 0.006 to 1.41) and older, during which the observed differences (in height) remains statistically significant (*P*<0.05). With regard to weight, no significant difference was observed until the age of 9 months (difference=379.62 gr, CI95%= 58.21 to 701.03) and older ages, during which the observed differences in weight are statistically significant. The pattern of growth in head circumference is also presented in Table 1. Accordingly, no significant difference was observed between the two groups in head circumference during the study period.

**Table 1:** Height, weight and head circumference differences between normal and stunted male children from birth to age 7

	Height (cm)			Weight (gr)			Head circumference (cm)		
Age	Difference between groups^a^	CI95%	*P*. value	Difference between groups	CI95%	*P*. value	Difference between groups	CI95%	*P*. value
Birth – 2 month (m)	0.37	(−0.25 to 0.99)	0.24	56.61	(−60.38 to 173.62)	0.34	−0.23	(− 1.02 to 0.55)	0.55
2–4 m	0.47	(−0.26 to 1.22)	0.20	118.19	(−95.41 to 331.81)	0.27	0.06	(−0.38 to 0.50)	0.79
4–6 m	0.22	(−0.47 to 0.91)	0.52	45.79	(− 212.09 to 303.68)	0.72	−0.07	(− 0.52 to 0.36)	0.72
6–9 m	0.70	(0.006 to 1.41)	0.04	105.54	(− 139.16 to 350.26)	0.39	−0.11	(− 0.52 to 0.28)	0.56
9–12 m	1.22	(0.45 to 1.99)	0.002	379.62	(58.21 to 701.03)	0.02	0.06	(− 0.35 to 0.48)	0.75
1–2 year (y)	1.87	(1.09 to 2.64)	< 0.001	438.50	(135.82 to 741.17)	0.005	−0.002	(−0.40 to 0.39)	0.98
2–3 y	3.91	(2.79 to 5.03)	< 0.001	726.65	(265.57 to 1187.74)	0.002			
3–4 y	4.83	(3.51 to 6.14)	< 0.001	854.88	(395.02 to 1314.75)	<0.001			
4–5 y	5.89	(3.39 to 8.40)	<0.001	1384.50	(824.00 to 1945.01)	<0.001			
5–6 y	7.95	(6.58 to 9.32)	<0.001	1384.98	(672.85 to 2097.10)	<0.001			
6–7 y	8.77	(7.60 to 9.94)	<0.001	1606.14	(836.09 to 2376.19)	<0.001			
7 y	9.38	(8.45 to 10.32)	<0.001	1265.19	(502.56 to 2027.82)	0.001			

^a^Normal – stunt children

Height, weight and head circumference of girls are compared between the two groups and the results are presented in Table 2. With regard to height, the observed difference remains relatively constant until 9 months (difference=0.97 cm, CI95%= 0.20 to 1.73) and older, during which the differences become predominantly significant (*P*<0.05). According to the results, height and head circumference of girls at birth are similar between the stunted and normal groups. However, significant differences in weight were observed from birth to 6 months of age in the benefit of the stunted group (*P*<0.05). The benefit turns to non-significant at the age of 9 months and onward (*P*>0.05). In addition, in favor of the stunted group, a constant and marginally significant difference was observed between the two groups in head circumference during the whole study period.

**Table 2:** height, weight and head circumference differences between normal and stunted female children from birth to age 7 y

	Height (cm)			Weight (gr)			Head circumference (cm)		
Age (month)	Difference between groups	CI95%	*P*. v	Difference between groups	CI95%	*P*. v	Difference between groups	CI95%	*P*. v
Birth – 2 month (m)	−0.53	(− 1.68 to 0.62)	0.36	− 142.96	(− 271.83 to − 14.08)	0.03	−0.79	(− 1.56 to − 0.01)	0.04
2–4 m	− 0.72	(−2.01 to 0.56)	0.26	− 234.37	(− 416.93 to − 51.81)	0.01	− 0.59	(− 1.42 to 0.22)	0.15
4–6 m	− 0.68	(− 2.06 to 0.70)	0.33	− 311.09	(− 569.99 to − 52.19)	0.01	−0.79	(− 1.62 to 0.03)	0.06
6–9 m	0.09	(−1.34 to 1.54)	0.89	--289.40	(− 556.27 to − 22.52)	0.03	−0.81	(− 1.66 to 0.036)	0.06
9–12 m	0.97	(0.20 to 1.73)	0.01	− 247.71	(− 561.41 to 65.98)	0.12	−0.31	(− 0.67 to 0.05)	0.09
1–2 year (y)	0.61	(−0.18 to 1.41)	0.13	−137.19	(− 450.52 to 176.13)	0.38	−0.17	(−0.51 to 0.17)	0.32
2–3 y	0.93	(−0.11 to 1.98)	0.08	− 181.43	(− 586.30 to 223.43)	0.37			
3–4 y	1.90	(0.71 to 3.09)	0.002	−18.57	(− 544.46 to 507.31)	0.94			
4–5 y	3.10	(1.85 to 4.35)	<0.001	87.11	(− 465.52 to 639.74)	0.75			
5–6 y	5.64	(4.46 to 6.82)	< 0.001	− 14.06	(− 670.00 to 641.88)	0.96			
6–7 y	7.01	(5.97 to 8.05)	< 0.001	119.27	(− 631.02 to 869.56)	0.75			
7 y	8.11	(7.24 to 8.97)	< 0.001	283.21	(− 457.54 to 1023.97)	0.45			

**Fig. 1:**
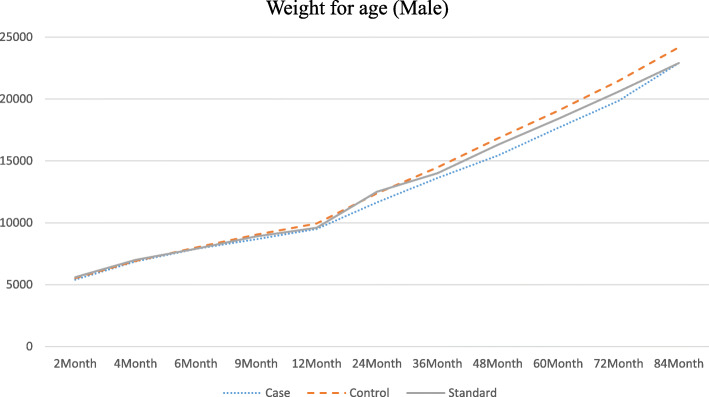
Weight (gr) for age among stunted and normal male participants from birth to age 84 months (7 years)

**Fig. 2:**
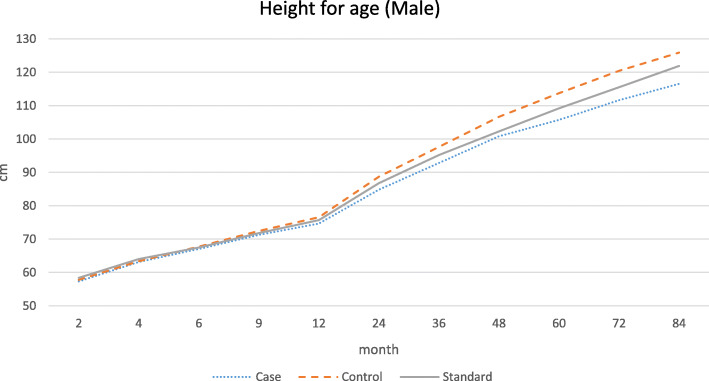
Height (cm) for age among stunted and normal male participants from birth to age 84 months (7 years)

**Fig. 3:**
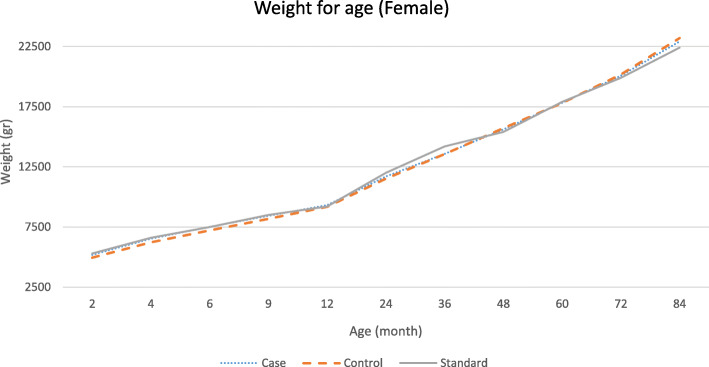
Weight (gr) for age among stunted and normal female participants from birth to age 84 months (7 years)

**Fig. 4:**
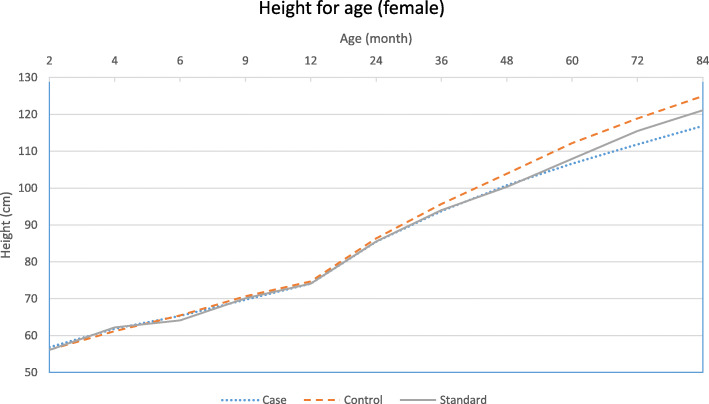
Height (cm) for age among stunted and normal female participants from birth to age 84 months (7 years)

The graphical patterns of the growth of the study groups for each gender are separately presented in Figs. 1, 2, 3 and 4. According to the Fig. 1, in male participants, regarding weight, the growth retardation started at about the age of 6 months with a relatively constant rate of deterioration till the end of the follow-up period. However, according to Fig. 2 when looking at height, a higher rate of diversion has been started since 48 months (4 years) of age onward. As presented in Figs. 3 and 4, growth retardation for girls is to some degree different from boys. First, it doesn’t seem that weight gaining is affected by stunting among girls. Also, retardation in linear growth seems to start later (9 months) among girls than boys with no apparent change in its rate then after. The figures reveal that growth retardation starts a couple of months later among girls compared to boys. For better illustration, the standard growth patterns for weight and height are also presented in the figures to make the conclusion easier.

## Discussion

Although there are socio-demographic diversities between different parts of the country, it seems that the stature of Iranian children has been improving over the past decades [18]. For example, results of a study in Northern Iran suggested that prevalence of stunting has been reduced from 32.8% (95% Cl; 31.0–34.6) in 1998 to 15.7% (95% Cl;14.3–17.2) in 2013 [19]. However, results of several studies suggested that short stature is still a major health problem in Iran [20, 21]. This study is conducted to understand the timing of stunting among boys and girls. Defining the starting point of stunting is a critical step in the detection and prevention of growth retardation.

Results of a pooled data analysis demonstrated significant benefits of exclusive breastfeeding on morbidity and mortality among children, [22, 23]. During infancy, several issues cause major concerns regarding the growth of a child. These include genetic deformities, environmental hazards and nutritional factors [24]. The recruited infants in the current population-based cohort study were all apparently healthy with normal anthropometric indexes at birth. This was done to minimize the effects of genetic factors and factors related to the antenatal period. Results from the first phase of our study revealed a number of factors altering the chance of stunting among the study population. Accordingly, diet (i.e. consumption of animal protein and dairy products and duration of breastfeeding) and socio-economic factors (i.e. family income and mother’s occupation) were reported to be effective in linear growth [14]. The current study aimed to define the age of the participants at which the retardation (stunting) starts. Also, to make a distinction between the patterns of growth among stunted and normal children in each gender, three important anthropometric measures from birth to the age of 7 (12 measures for height and weight and 6 measures for head circumference) were included in the analysis. The results revealed a very important phenomenon in the linear growth of the stunted children, i.e. stunted boys and girls, experienced a significant diversion from the linear growth of the normal children (and the corresponding standard growth chart) at about 6 months and 9 months of age respectively. However, as presented in the results section, the time of shifting in growth seems similar in boys and girls, as on average, the stunted girls were taller than normal children from birth to 6^th^ month of age. At this age, the stunted children started to become shorter than the normal children. At about six months of age, children start experiencing important changes such as, start weaning from breast milk and start eating supplementary and more solid foods.

According to the present study, the estimated prevalence of stunting among the study population was about 18%. The prevalence of stunting in Iranian children and its associated factors have been addressed in several studies [18, 25–27], However, these studies did not pay any attention to the temporal pattern of stunting and the approximate age of stunting among the Iranian children. For example, a systematic review and meta-analysis on the prevalence of stunting in Iranian children under 5-years of age estimated that the pooled prevalence of stunting is 12% (95%CI: 10–14) and that the prevalence of stunting in boys (10%) and girls (9%) are equal [26]. The review also suggested that the overall prevalence of stunting in Southern provinces (including Fars) of Iran is mostly higher (17%) than in western (9%), eastern (8%), southern (17%) and central regions (15%) [26]. However, this review did not examine the reasons for these discrepancies and the age at which the faltering of linear growth is started.

Results of a study on the prevalence of stunting in 3147 school children from 5 districts of Tehran suggested that only 4% of the study participants were defined as stunted. The authors concluded that birth weight, maternal age and fathers’ height are the major contributing factors in stunting among the study population. However, the study provided no stratification analysis by sex and timing of growth retardation [18]. Emamian et. al., suggested a considerable socio-economic inequality in stunting among the Iranian population and that maternal education was the most important factor associated with stunting among children under 6 years [27]. Again, no timing for stunting was reported.

A study in rural Guatemalan children shows that growth faltering starts soon after birth. Authors suggested that at 3 year of age, growth-retarded children were on average 3.6 kg lighter than the WHO/CDC growth chart. Also, between 19% and 34% of the deficit at 3 years of age was due to failure to thrive during the first 3 months of life, 12% to 19% between 3 and 6 months and 12% to 25% between 6 and 9 months of age [28]. However, the authors did not report the status of the linear growth of the study population.

Results of a population-based cohort study in sub-Saharan Africa provided evidence that retardation of linear growth among children under 5 years of age started soon after birth and continued throughout infancy [29]. The authors suggested that among several factors considered in the study (including; complementary feeding, morbidity, maternal short stature and gender) the strongest predictor of severe stunting at 12 months of age was small birth size [29]. Another cohort study has suggested that retardation in linear growth among Zambian infants is more sever at about 13 months of age. According to the authors, the suggested age is about the time which nearly all the infants have weaned off breast milk a factor which may have severely affect children’s growth [24].

The results of the current study also revealed another age at which growth rate becomes smaller among stunted children and even more diversion from normal growth is observed (about 24 months of age). At this age, children start walking, touching objects, go outside home and more importantly eat family foods. Interestingly, the reduced growth rate continues for girls to the end of the follow-up period but for boys, another major reduction occurs at the fourth year of age. At this age, boys start more active playing with friends or other siblings, whereas girls do sedentary plays [30]. That is why, as presented in the figures, weight for age among girls of both stunted and normal groups is higher than the corresponding standard weight for age of the same gender.

### Strength and limitations

The participants in the present study were selected randomly among all children in the defined population. Participant’s growth indexes were followed from birth until 7 years of age. All children had normal height and weight at birth helping us to control for genetic and antenatal related factors affecting growth. All information regarding the growth and diet of the participants was collected from the participant’s health file. Also, Therefore, major sources of recall or reporting bias are not expected to exist. However, as some information collected from the phone interview, this may prone to recall and information bias, that may affect the results. To tackle this recall and reporting biases, mothers were not told why they were selected (whether if their child is a case or control). Additionally, no detailed information was available for the supplementary foods and adequacy of breast milk. Lastly, as the main aim of this study was to find approximate age at which stunting starts among boys and girls, we did not examined the prevalence of severe, moderate and mild stunting in both genders, which we recommend to be examined in the further research on stunting.

## Conclusions

Iranian children are mainly born with a normal weight and height, which suggest a normal growth during the antenatal period (or pregnancy of mother). However, after birth (at about 6 months of age) the growth pattern of some children starts to stumble and divert from the normal pattern. The similarity in the timing of stunting in girls and boys suggests a similarity in the contributing factors, which (according to the results of the previous study) are mainly modifiable (e.g. mother’s involvement in child’s daily care, the timing of weaning breast milk and types of diet including supplementary foods). In addition, the higher weight for age among the normal and stunted children compared to the standard figures at age 7 suggests that a high energy-low protein diet may play important roles in the growth pattern of the study population. With regard to the results of the previous and current studies, the approaches of Iranian mothers to the breast feeding and weaning should be observed closely to understand how change to a diet during infancy can affect a child’s growth. The results raise the importance of providing families with proper knowledge and cost-effective practices in the timing and methods of weaning and starting supplementary foods. We could not conclude whether growth-retarded children are able to catch up if the contributing factors are corrected. As a result, a specially designed study is needed to understand the reasons for observing such a pattern in growth and its associates among Iranian children.

## Data Availability

The datasets used and analyzed during the current study are available from the corresponding author on reasonable request.

## Abbreviations

WHO: World Health Organization
NCHS: National Center for Health Statistics
OR: Odds Ratio
95% CI: 95% Confidence Interval
